# Coronary risk in relation to genetic variation in *MEOX2* and *TCF15* in a Flemish population

**DOI:** 10.1186/s12863-015-0272-2

**Published:** 2015-10-01

**Authors:** Wen-Yi Yang, Thibault Petit, Lutgarde Thijs, Zhen-Yu Zhang, Lotte Jacobs, Azusa Hara, Fang-Fei Wei, Erika Salvi, Lorena Citterio, Simona Delli Carpini, Yu-Mei Gu, Judita Knez, Nicholas Cauwenberghs, Matteo Barcella, Cristina Barlassina, Paolo Manunta, Giulia Coppiello, Xabier L. Aranguren, Tatiana Kuznetsova, Daniele Cusi, Peter Verhamme, Aernout Luttun, Jan A. Staessen

**Affiliations:** Research Unit Hypertension and Cardiovascular Epidemiology, KU Leuven Department of Cardiovascular Sciences, University of Leuven, Kapucijnenvoer 35, Box 7001, BE-3000 Leuven, Belgium; Cardiology, Department of Cardiovascular Sciences, University of Leuven, Leuven, Belgium; Genomics and Bioinformatics Platform at Filarete Foundation, Department of Health Sciences and Graduate School of Nephrology, Division of Nephrology, San Paolo Hospital, University of Milan, Milan, Italy; Division of Nephrology and Dialysis, IRCCS San Raffaele Scientific Institute, University Vita-Salute San Raffaele, Milan, Italy; School of Nephrology, University Vita-Salute San Raffaele, Milan, Italy; Centre for Molecular and Vascular Biology, Department of Cardiovascular Sciences, University of Leuven, Leuven, Belgium; R & D VitaK Group, Maastricht University, Maastricht, The Netherlands

**Keywords:** Clinical genetics, Coronary heart disease, *MEOX2*, Population science, *TCF15*, Translational research

## Abstract

**Background:**

In mice MEOX2/TCF15 heterodimers are highly expressed in heart endothelial cells and are involved in the transcriptional regulation of lipid transport. In a general population, we investigated whether genetic variation in these genes predicted coronary heart disease (CHD).

**Results:**

In 2027 participants randomly recruited from a Flemish population (51.0 % women; mean age 43.6 years), we genotyped six SNPs in *MEOX2* and four in *TCF15*. Over 15.2 years (median), CHD, myocardial infarction, coronary revascularisation and ischaemic cardiomyopathy occurred in 106, 53, 78 and 22 participants. For SNPs, we contrasted CHD risk in minor-allele heterozygotes and homozygotes (variant) *vs.* major-allele homozygotes (reference) and for haplotypes carriers (variant) *vs.* non-carriers. In multivariable-adjusted analyses with correction for multiple testing, CHD risk was associated with *MEOX2* SNPs (*P* ≤ 0.049), but not with *TCF15* SNPs (*P* ≥ 0.29). The *MEOX2 GTCCGC* haplotype (frequency 16.5 %) was associated with the sex- and age-standardised CHD incidence (5.26 *vs*. 3.03 events per 1000 person-years; *P* = 0.036); the multivariable-adjusted hazard ratio [HR] of CHD was 1.78 (95 % confidence interval, 1.25–2.56; *P* = 0.0054). For myocardial infarction, coronary revascularisation, and ischaemic cardiomyopathy, the corresponding HRs were 1.96 (1.16–3.31), 1.87 (1.20–2.91) and 3.16 (1.41–7.09), respectively. The *MEOX2 GTCCGC* haplotype significantly improved the prediction of CHD over and beyond traditional risk factors and was associated with similar population-attributable risk as smoking (18.7 % *vs.* 16.2 %).

**Conclusions:**

Genetic variation in *MEOX2*, but not *TCF15*, is a strong predictor of CHD. Further experimental studies should elucidate the underlying molecular mechanisms.

**Electronic supplementary material:**

The online version of this article (doi:10.1186/s12863-015-0272-2) contains supplementary material, which is available to authorized users.

## Background

Endothelial cells lining the microvasculature constitute the interface between the circulating blood and tissues [1]. They differentiate to acquire the molecular, morphological and functional characteristics required for proper organ function [1]. In the heart, endothelial cells play an active role in the transport of fatty acids, the principal energy source for the continuously beating muscle [1, 2]. Using microarray profiling on endothelial cells isolated from the heart, brain, and liver of mice, we recently identified a specific genetic signature for heart endothelial cells, including MEOX2/TCF15 heterodimers as novel transcriptional determinants [3]. This signature was largely shared with skeletal muscle and adipose tissue endothelium and was enriched in genes encoding fatty acid transport-related proteins [3]. Using gain- and loss-of-function approaches, we showed that MEOX2/TCF15 mediates fatty acid uptake in heart endothelial cells, in part, by driving endothelial CD36 and lipoprotein lipase (LPL) expression and thereby facilitating fatty acid transport across cardiac endothelial cells [3].

LPL is expressed at the luminal endothelial surface of arteries and capillaries and hydrolyses circulating lipoprotein triglycerides into free fatty acids and glycerol [4]. Local [4] and systemic [5] dysregulation of lipid metabolism and endothelial dysfunction [6, 7] are hallmarks of coronary atherosclerosis and long precede clinically overt disease. These observations suggest that genetic predisposition plays an important role in the pathogenesis of coronary heart disease (CHD) [6, 7]. In view of our recent observations of enriched expression of MEOX2 and TCF15 in heart endothelial cells [3], we hypothesised that genetic variation in the genes encoding these transcription factors might be associated with coronary risk. To test this hypothesis, we analysed data accumulated since 1985 in a Flemish population study [8, 9].

## Methods

### Study population

The Flemish Study on Environment, Genes and Health Outcomes (FLEMENGHO) complies with the Helsinki declaration for research in human subjects and the Belgian legislation for the protection of privacy (http://www.privacycommission.be). The Ethics Committee of the University of Leuven approved the study. Recruitment for the FLEMENGHO study started in 1985 [8, 9]. From August 1985 to November 1990, a random sample of the households living in a geographically defined area of Northern Belgium was investigated with the goal to recruit an equal number of participants in each of six strata by sex and age (20–39, 40–59, and ≥60 years). All household members aged 20 years or older were invited, if the quota of their sex-age group had not yet been met. From June 1996 until January 2004 recruitment of families continued using the former participants (1985–1990) as index persons and including teenagers. The participants were repeatedly followed up. In all study phases, we used the same standardised methods to measure blood pressure and to administer questionnaires. The participation rate at enrolment was 78.0 %. At each contact, participants gave or renewed informed written consent.

Of 3343 enrolled participants, we excluded 1316 from analysis, because blood stored in the biobank was exhausted with no material left for genotyping (*n* = 521), because of DNA degradation (*n* = 314), because at enrolment they were less than 20 years old (*n* = 372), or because one or more of the six *MEOX2* or four *TCF15* SNPs were unavailable (*n* = 109). Thus, the number of participants statistically analysed totalled 2027.

### Measurements at baseline

Trained nurses measured the participants’ anthropometric characteristics and blood pressure. Body mass index was weight in kilograms divided by the square of height in meters. Blood pressure was the average of five consecutive auscultatory readings obtained with a standard mercury sphygmomanometer after participants had rested in the sitting position for at least 5 min. Hypertension was a blood pressure of at least 140 mm Hg systolic or 90 mm Hg diastolic, or use of antihypertensive drugs. The nurses also administered a standardised questionnaire inquiring about each participant’s medical history, smoking and drinking habits, and intake of medications. Plasma glucose and serum total and high-density lipoprotein (HDL) cholesterol and serum creatinine were measured by automated methods in certified laboratories. Diabetes mellitus was a fasting or random plasma glucose level exceeding 7.0 or 11.1 mmol/L, or use of antidiabetic agents [10].

### Ascertainment of coronary events

FLEMENGHO received ethical approval. The database was registered with the Privacy Commission. These legal requirements being fulfilled, we could ascertain the vital status of participants at annual intervals until 06 December 2012 via the Belgian Population Registry. In addition, we could obtain the International Classification of Disease codes for the immediate and underlying causes of death from the Flemish Registry of Death Certificates. For 1853 participants, we collected information on the incidence of non-fatal endpoints either via face-to-face follow-up visits with repeated administration of the same standardised questionnaire as used at baseline (*n* = 1521) or via a structured telephone interview (*n* = 332). Follow-up data were available from one visit in 360 participants, from two in 304, from three in 436, and from four or more in 421 participants.

Trained nurses used the International Classification of Diseases to code incident cases of CHD. Two investigators blinded with regard to the genotypic results adjudicated all coronary events against the medical records of general practitioners or hospitals. Coronary events included sudden death, fatal and non-fatal myocardial infarction, acute coronary syndrome requiring hospitalisation, ischaemic cardiomyopathy, and surgical or percutaneous coronary revascularisation. In the outcome analyses, we only considered the first event within each category.

### Genotyping

Ethics approval and informed consent covered genotyping. After DNA extraction from peripheral blood [11], SNPs were genotyped using the TaqMan® OpenArray™ Genotyping System (Life Technologies, Foster City, CA). All DNA samples were loaded at 50 ng/mL and amplified according to the manufacturer’s instructions. For analysis of the genotypes, we used autocalling methods, as implemented in the TaqMan Genotyper software version 1.3 (Life Technologies). Next, genotype clusters were evaluated manually with the sample call rate set above 0.90. Sixteen duplicate samples gave 100 % reproducibility for all 64 SNPs on the custom made array, including the genes of interest in the current article [12].

*MEOX2* (75601 base pairs) maps to chromosome 7 (p22.1–p21.3). To select the *MEOX2* SNPs to genotype, we first reviewed all SNPs in this gene, including the flanking regions, as available in the Illumina 1 M Duo and OmniExpress arrays (San Diego, CA). We excluded SNPs with a minor allele frequency of less than 1 % and those that were in high linkage disequilibrium (*r*^*2*^ ≥ 0.80). Next, based on the availability of SNPs on the TaqMan OpenArray Genotyping System, we selected 12 tagging SNPs (rs6946099, rs10777, rs7800473, rs13438001, rs12056299, rs7787043, rs758297, rs4532497, rs10263561, rs6959056, rs740566, rs1050290) that are in high linkage disequilibrium (*r*^*2*^ ≥ 0.80) with 92 neighbouring SNPs (Additional file 1: Figure S1 and Table S1), but were not in high linkage disequilibrium (*r*^*2*^ < 0.80) with one another. The 12 selected SNPs covered the entire gene with extension into the 3’ and 5’ flanking regions. We excluded six SNPs with a successful genotyping call rate of less than 0.98. Finally, we retained six *MEOX2* SNPs (rs10777, rs12056299, rs7787043, rs4532497, rs6959056, and rs1050290) in the analysis (Additional file 1: Table S2) that are in linkage disequilibrium (*r*^*2*^ > 0.80) with 23 other SNPs (Additional file 1: Table S1). *TCF15* (6602 base pairs) maps to chromosome 20p13. We genotyped five SNPs covering the whole gene (rs282152, rs6116745, rs282162, rs3761308 and rs12624577), but excluded rs282152, because the SNP call rate was less than 0.98 (Additional file 1: Figure S2).

### Statistical analysis

For database management and statistical analysis, we used SAS software, version 9.3 (SAS Institute, Cary, NC). For comparison of means and proportions, we applied the large sample z-test or ANOVA and Fisher’s exact, respectively. We tested Hardy-Weinberg equilibrium in unrelated founders, using the exact statistics available in the PROC ALLELE procedure of the SAS package. For analysis of single SNPs, we combined the least frequent homozygous group with heterozygous subjects. We tested linkage disequilibrium and reconstructed haplotypes using the SAS procedures PROC ALLELE and PROC HAPLOTYPE. To check for consistency, we repeated haplotype construction accounting for pedigree information using SHAPEIT version 2 (http://mathgen.stats.ox.ac.uk/genetics_software/shapeit/shapeit.html [13]).

We compared the incidence of coronary endpoints in relation to genetic variants, using (i) rates standardised by the direct method for sex and age (<40, 40–59, ≥60 years) and (ii) the cumulative incidence derived from Cox models adjusted for sex and age. Next, we assessed the prognostic value of the genetic variants in multivariable-adjusted Cox regression. We checked the proportional hazard assumption by applying a Kolmogorov-type supremum test as implemented in the ASSESS statement of the PROC PHREG procedure. To account for family clusters, we used the PROC SURVIVAL procedure of the SUDAAN 11.0.1 software (Research Triangle Institute, NC). In this procedure, non-independence among family members was taken into account by including family as a random effect along with other covariables as fixed effects. We analysed genotypes and haplotypes using major allele homozygotes and non-carriers as the reference group, respectively. We adjusted *P* values for the associations between outcomes and genetic variants, using the Benjamini and Hochberg false discovery rate [14] according to the number of SNPs retained in the analysis.

We computed the positive predictive value of the risk carrying *MEOX2* haplotype *GTCCGC* as (R × D)/([G/100] × [R −1] + 1), where R is the multivariable-adjusted hazard ratio, D is the incidence of CHD in the whole population, and G is the prevalence of the *GTCCGC* haplotype [15]. The attributable risk is given by ([R −1] × 100)/R and the population-attributable risk by ([G/100] × [R −1] × 100)/([G/100] × [R −1] + 1) [15]. Finally, we assessed the power of the *MEOX2 GTCCGC* haplotype to predict CHD over and beyond classical risk factors, using the integrated discrimination improvement (IDI) and the net reclassification improvement (NRI), as described by Pencina and colleagues for survival data [16].

## Results

### Baseline characteristics

All 2027 participants were White Europeans, of whom 1034 (51.0 %) were women. The study population consisted of 332 singletons and 1695 related subjects, belonging to 49 single-generation families and 191 multi-generation pedigrees. Age averaged (±SD) 43.6 ± 14.3 years, blood pressure 125.0 ± 15.4 mm Hg systolic and 76.2 ± 9.5 mm Hg diastolic, body mass index 25.7 ± 4.3 kg/m^2^, and total cholesterol 5.49 ± 1.15 mmol/L. Among all participants, 486 (24.0 %) had hypertension, of whom 214 (44.0 %) were on antihypertensive drug treatment, 33 (1.6 %) had diabetes mellitus, and 41 (2.0 %) reported a history of CHD. Previous coronary complications included angiographically proven coronary stenosis, myocardial infarction, and coronary revascularisation in 8 (0.4 %), 11 (0.5 %) and 22 (1.1 %) patients, respectively. Of 1034 women and 993 men, 277 (26.8 %) women and 328 (33.0 %) men were smokers, and 168 (16.3 %) women and 418 (42.1 %) men reported intake of alcohol. In smokers, median tobacco use was 15 cigarettes per day (interquartile range, 10 to 20 cigarettes per day). In drinkers, the median alcohol consumption was 14 g per day (8 to 26 g per day).

Table 1 lists the baseline characteristics of participants according to CHD incidence. Most risk factors differed in the expected direction between cases and non-cases. However, compared with non-cases, the prevalence of smoking was not different in patients with incident CHD (29.6 % *vs.* 36.8 %; *P* = 0.13), while the prevalence of drinking was lower among cases (29.4 % *vs.* 19.8 %; *P* = 0.036). Heart rate at baseline was similar in participants without and with incident CHD (69.2 *vs.* 69.9 beats per minute; *P* = 0.43).

**Table 1:** Baseline characteristics of participants by incident CHD

Characteristic	Non-cases	Cases	All
N°	1921	106	2027
N° with characteristics (%)			
Women	1006 (52.4)	28 (26.4)‡	1034 (51.0)
Current smoker	566 (29.6)	39 (36.8)	605 (29.9)
Drinking alcohol	565 (29.4)	21 (19.8)*	586 (28.9)
Diabetes mellitus	26 (1.4)	7 (6.6)‡	33 (1.6)
Hypertension	432 (22.5)	54 (50.9)‡	486 (24.0)
Treated hypertension	189 (9.8)	25 (23.6)‡	214 (10.6)
History of CHD	30 (1.6)	11 (10.4)‡	41 (2.0)
Mean of characteristic (±SD)			
Age, years	42.8 ± 14.1	57.4 ± 11.3‡	43.6 ± 14.3
Body mass index, kg/m^2^	25.6 ± 4.3	27.1 ± 3.9‡	25.7 ± 4.3
Waist-to-hip ratio	0.84 ± 0.09	0.91 ± 0.08‡	0.85 ± 0.09
Systolic blood pressure, mm Hg	124.5 ± 15.2	135.3 ± 15.8‡	125.0 ± 15.4
Diastolic blood pressure, mm Hg	76.0 ± 9.4	78.6 ± 9.9†	76.2 ± 9.5
Heart rate, beats per minute	69.2 ± 9.5	69.9 ± 9.0	69.3 ± 9.5
Total cholesterol, mmol/L	5.46 ± 1.14	6.06 ± 1.15‡	5.49 ± 1.15
HDL cholesterol, mmol/L	1.40 ± 0.39	1.16 ± 0.33‡	1.37 ± 0.39
Total-to-HDL cholesterol ratio	4.21 ± 1.58	5.75 ± 2.30‡	4.29 ± 1.66
Serum creatinine, μmol/L	90.2 ± 16.8	104.3 ± 19.4‡	91.1 ± 16.8
Plasma glucose, mmol/L	5.03 ± 1.27	5.48 ± 2.29‡	5.05 ± 1.35

HDL cholesterol refers to the serum concentration of high-density lipoprotein cholesterol. Diabetes mellitus was a fasting or random plasma glucose level exceeding 7.0 or 11.1 mmol/L, or use of antidiabetic agents. Hypertension was a blood pressure of ≥140 mm Hg systolic or ≥90 mm Hg diastolic or use of antihypertensive drugs. Significance of the differences between non-cases and cases: * *p* ≤ 0.05; † *p* ≤ 0.01; ‡ *p* ≤ 0.001

### Incidence of events

Over a median follow-up of 15.2 years (5th to 95th percentile interval, 5.7 to 27.1 years), 106 new coronary events occurred, 24 fatal and 82 non-fatal. Coronary events comprised 12 fatal and 34 non-fatal myocardial infarcts and 7 sudden deaths. There were 78 patients who underwent surgical (*n* = 29) or percutaneous (*n* = 56) coronary revascularisation. Coronary events also included 5 fatal and 17 non-fatal cases of ischaemic cardiomyopathy.

### Analyses of single SNPs in *MEOX2* and *TCF15*

Additional file 1: Table S2 describes the position and the SNPs retained in the analysis and the allele and genotype frequencies in 825 unrelated founders. The six SNPs in *MEOX2* and the four SNPs in *TCF15* complied with Hardy-Weinberg equilibrium (0.30 ≤ *P* ≤ 0.80). In the whole study population (Additional file 1: Table S3), the frequencies of the minor alleles ranged from 21.1 to 43.0 % for *MEOX2* and from 9.6 to 38.5 % for *TCF15*. The prevalence of minor allele homozygotes ranged from 4.4 to 17.8 % for *MEOX2*, and from 0.7 to 15.9 % for *TCF15*.

**Fig. 1:**
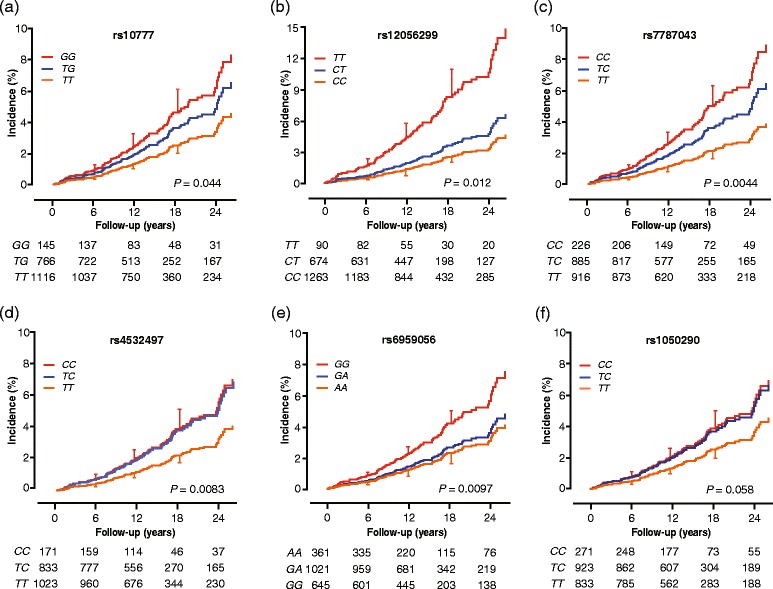
CHD Incidence by genotype for six *MEOX2* SNPs (Panels **a**-**f**). Estimates were standardised to the mean of the distributions of sex and age in the whole study population. Vertical bars denote the standard error. *P*-values refer to the difference between minor allele carriers and major allele homozygotes. Cumulative incidence did not differ between minor allele homozygotes and heterozygotes (0.23 ≤ *P* ≤ 0.98 [**a**, **c**-**f**]), except for rs12056299 (*P* = 0.014 [**b**]). Median follow-up was 15.2 years. Tabulated data are the number of participants at risk by genotype at 6-year intervals

The sex- and age-standardised incidence rates of coronary events associated with the *MEOX2* SNPs appear in Additional file 1: Table S4. Compared with major allele homozygotes, minor allele carriers experienced a higher CHD incidence except for rs6959056. The sex- and age-adjusted cumulative incidence of coronary events (Fig. 1) showed significant association (*P* ≤ 0.012) with the *MEOX2* SNPs except for rs1050290 (*P* = 0.058). There were no differences in these estimates between homozygous and heterozygous minor allele carriers (0.23 ≤ *P* ≤ 0.98) except for rs12056299 (*P* = 0.014). For all coronary events combined, the sex- and age-standardised incidence rates (0.11 ≤ *P* ≤ 0.39) and the sex- and age-adjusted cumulative incidence (0.11 ≤ *P* ≤ 0.71) did not differ among minor allele carriers and major allele homozygotes of the four *TCF15* SNPs.

Next, we accounted for family clusters and adjusted the hazard ratios for baseline characteristics, including sex, age, body mass index, systolic pressure, the total-to-HDL cholesterol ratio, smoking and drinking, and antihypertensive drug treatment. Compared with homozygotes of the major allele, for rs10777, rs12056299, rs7787043, rs4532497, and rs1050290, CHD risk was higher in minor allele carriers, whereas the opposite was the case for rs6959056 (Table 2). These findings remained consistent with correction for multiple testing (Table 2) and after excluding patients who had a history of CHD at baseline (Additional file 1: Table S5).

**Table 2:** Multivariable-adjusted hazard ratios for CHD by *MEOX2* SNPs

SNP Event	N° events/at risk		Hazard ratio	*P*	*P*_BH_
	Minor allele carriers	Major allele homozygotes			
rs10777	***GG + TG***	***TT***			
All coronary events	59/911	47/1116	1.56 (1.06–2.29)	0.024	0.029
Myocardial infarction	31	22	1.75 (1.01–3.03)	0.045	0.054
Coronary revascularisation	42	36	1.42 (0.90–2.24)	0.13	0.14
Ischaemic cardiomyopathy	16	6	4.68 (1.86–11.77)	0.0011	0.0066
rs12056299	***TT + CT***	***CC***			
All coronary events	51/764	55/1263	1.68 (1.17–2.41)	0.0052	0.016
Myocardial infarction	28	25	2.05 (1.20–3.51)	0.0087	0.042
Coronary revascularisation	37	41	1.59 (1.02–2.49)	0.041	0.082
Ischaemic cardiomyopathy	14	8	3.37 (1.49–7.62)	0.0036	0.011
rs7787043	***CC + TC***	***TT***			
All coronary events	74/1111	32/916	1.72 (1.13–2.62)	0.011	0.023
Myocardial infarction	38	15	1.82 (1.02–3.27)	0.044	0.054
Coronary revascularisation	54	24	1.68 (1.02–2.77)	0.041	0.082
Ischaemic cardiomyopathy	16	6	2.22 (0.84–5.86)	0.11	0.20
rs4532497	***CC + TC***	***TT***			
All coronary events	66/1004	40/1023	1.80 (1.22–2.66)	0.0031	0.016
Myocardial infarction	33	20	1.88 (1.03–3.42)	0.040	0.054
Coronary revascularisation	50	28	1.88 (1.20–2.96)	0.0062	0.037
Ischaemic cardiomyopathy	14	8	1.97 (0.80–4.83)	0.14	0.20
rs6959056	***AA + GA***	***GG***			
All coronary events	59/1382	47/645	0.62 (0.42–0.92)	0.017	0.025
Myocardial infarction	27	26	0.52 (0.31–0.88)	0.014	0.042
Coronary revascularisation	46	32	0.71 (0.45–1.12)	0.14	0.14
Ischaemic cardiomyopathy	12	10	0.75 (0.33–1.70)	0.49	0.49
rs1050290	***CC + TC***	***TT***			
All coronary events	73/1194	33/833	1.50 (1.00–2.26)	0.049	0.049
Myocardial infarction	36	17	1.48 (0.81–2.70)	0.20	0.20
Coronary revascularisation	55	23	1.59 (0.99–2.56)	0.055	0.083
Ischaemic cardiomyopathy	16	6	1.97 (0.75–5.16)	0.17	0.20

Numbers of events do not add up, because only the first event in each category was analysed. Hazard ratios (95 % confidence interval) express the risk of minor allele carriers *vs.* major allele homozygotes, account for family clusters, and were adjusted for baseline characteristics including sex, age, body mass index, systolic pressure, total-to-HDL cholesterol ratio, smoking and drinking, and antihypertensive drug treatment. *P* and *P*_BH_ indicate the significance of the hazard ratios without and with Benjamini-Hochberg’s correction for multiple testing

For the four *TCF15* SNPs, the multivariable-adjusted hazard ratios modelling the CHD risk of minor allele carriers *vs.* major allele homozygotes did not reach significance (0.75 ≤ hazard ratio ≤ 1.45; 0.072 ≤ *P* ≤ 0.52). However, Additional file 1: Figure S3 shows interaction (*P* = 0.011) between rs12624577 in *TCF15* and rs4532497 in *MEOX2*. Among *C* allele carriers of *MEOX2* rs4532497, the hazard ratio expressing the risk of the *C* allele relative to the *TT* genotype in *TCF15* was 2.44 (95 % confidence interval, 1.38–4.29; *P* = 0.0021), whereas among MEOX2 *TT* homozygotes the corresponding hazard ratio was 0.75 (0.37–1.51; *P* = 0.42).

### Analysis of *MEOX2* haplotypes

Using the expectation-maximisation algorithm as implemented in the PROC HAPLOTYPE procedure of the SAS software version 9.3, three haplotypes of *MEOX2* SNPs had a frequency of over 10 % and were carried through in the analysis. With letters referring to the alleles in rs10777, rs12056299, rs7787043, rs4532497, rs6959056, and rs1050290 in *MEOX2*, these haplotypes were *TCTTAT* (27.5 %), *TCTTGT* (26.4 %), and *GTCCGC* (16.5 %). For all coronary events, rates standardised for sex and age (5.26 *vs*. 3.03 events per 1000 person-years; Additional file 1: Table S6*)* and cumulative incidence estimates adjusted for sex and age (Additional file 1: Figure S4), both before (*P* ≤ 0.012) and after adjustment for multiple testing (*P* ≤ 0.036), showed significant association with *GTCCGC*. In multivariable-adjusted analyses (Table 3), *GTCCGC* carriers, compared to non-carriers, had a 78 % higher CHD risk. The *p*-values before and after correction for multiple testing were 0.0018 and 0.0054, respectively. Myocardial infarction, coronary revascularisation and ischaemic cardiomyopathy all showed significant association with the *GTCCGC* haplotype. These findings were not materially different when we reconstructed haplotypes accounting for pedigree information (Additional file 1: Table S7) or after excluding patients who had a history of CHD at baseline (Additional file 1: Table S8).

**Table 3:** Multivariable-adjusted hazard ratios for CHD by *MEOX2* haplotypes

Haplotype Event	N° events/at risk		Hazard ratio	*P*	*P*_BH_
	Carrier	Non-carrier			
*TCTTAT*					
All coronary events	40/951	66/1076	0.73 (0.49–1.11)	0.14	0.21
Myocardial infarction	20	33	0.77 (0.42–1.41)	0.40	0.56
Coronary revascularisation	30	48	0.75 (0.47–1.19)	0.22	0.22
Ischaemic cardiomyopathy	6	16	0.56 (0.21–1.51)	0.25	0.25
*TCTTGT*					
All coronary events	46/937	60/1090	0.90 (0.58–1.39)	0.63	0.63
Myocardial infarction	26	27	1.19 (0.67–2.11)	0.56	0.56
Coronary revascularisation	30	48	0.74 (0.46–1.20)	0.22	0.22
Ischaemic cardiomyopathy	9	13	0.59 (0.24–1.43)	0.24	0.25
*GTCCGC*					
All coronary events	43/614	63/1413	1.78 (1.24–2.56)	0.0018	0.0054
Myocardial infarction	23	30	1.96 (1.16–3.31)	0.012	0.036
Coronary revascularisation	33	45	1.87 (1.20–2.91)	0.0058	0.017
Ischaemic cardiomyopathy	11	11	3.16 (1.41–7.09)	0.0053	0.016

Numbers of events do not add up, because only the first event in each category was analysed. Letters coding the haplotypes refer to the rs10777, rs12056299, rs7787043, rs4532497, rs6959056 and rs1050290 alleles (see Additional file 1: Table S1 and S2). Haplotypes were reconstructed using the expectation-maximisation algorithm as implemented in the PROC HAPLOTYPE procedure of the SAS software version 9.3. Hazard ratios (95 % confidence interval) express the risk associated with carrying *vs.* not carrying a haplotype, account for family clusters, and were adjusted for baseline characteristics including sex, age, body mass index, systolic pressure, total-to-HDL cholesterol ratio, smoking and drinking, and antihypertensive drug treatment. *P* and *P*_BH_ indicate the significance of the hazard ratios without and with Benjamini-Hochberg’s correction for multiple testing

With adjustments applied as before, the positive predictive value and the attributable and population-attributable CHD risks associated with *GTCCGC* were 7.5, 43.1 and 18.7 %, respectively*.* For smoking, analysed as reference, the corresponding estimates (unadjusted for genetic risk) were 7.2, 39.3 and 16.2 %, respectively*.* Table 4 shows that in all participants and in those without CHD at entry, adding the *GTCCGC* haplotype to the basic model including traditional risk factors improved (0.016 ≤ *P* ≤ 0.056) IDI and NRI.

**Table 4:** Improvement in predicting CHD events by adding haplotype *GTCCGC* to the basic model

Study group	Integrated discrimination improvement		Net reclassification improvement	
	%Δ (95 % CI)	*P*	%Δ (95 % CI)	*P*
All (*n* = 2027)	0.81 (−0.02 to 1.65)	0.056	21.7 (2.5 to 40.8)	0.026
Free of CHD at entry (*n* = 1986)	1.15 (0.17 to 2.12)	0.021	24.9 (4.7 to 45.3)	0.016

**%**Δ is the percentage change (95 % confidence interval). The basic model includes the baseline covariables sex, age, body mass index, systolic pressure, total-to-HDL cholesterol ratio, smoking and drinking, and antihypertensive drug treatment. The integrated discrimination improvement is the difference between the discrimination slopes of the basic model and the basic model extended with the *GTCCGC* haplotype. The discrimination slope is the difference in predicted probabilities between non-cases and cases. The net reclassification improvement is the sum of the percentages of participants correctly reclassified to non-cases and cases

## Discussion

To our knowledge, our study is the first to relate in a general population CHD incidence to genetic variation in *MEOX2* and *TCF15*, two transcription factors that are highly expressed by cardiac endothelium and that in a heterodimeric fashion interfere with cardiac energy metabolism by driving endothelial CD36 and LPL expression, thereby facilitating fatty acid transport across the cardiac endothelium [3]. The key finding of our current study was that the risk of advanced CHD was associated with genetic variation in *MEOX2*, as captured by six tagging SNPs. On the other hand, genetic variation in *TCF15*, coding for the heterodimeric partner of MEOX2, was not associated with the incidence of coronary events. Nonetheless, the CHD risk associated with *MEOX2* rs4532497 was confined to *TCF15* rs12624577 variant allele carriers, which might reflect the known heterodimeric action picked up in our experimental studies [3]. Although our current study firmly established an association between CHD risk and genetic variation in *MEOX2*, the molecular mechanisms underlying this relation need further clarification in experimental studies back translating our epidemiological findings. For now, working hypotheses might be developed along two lines respectively involving disturbed lipid handling [5, 17–19], a key mechanism in atherosclerosis, or the involvement of MEOX2 in the angiogenic responses to stressors [20–24] or in the migration or proliferation of endothelial and vascular smooth muscle cells [25, 26].

A large-scale genome-wide association study identified 46 significant lead SNPs associated with CHD. Twelve showed a significant association with a lipid trait. Variation in the *MEOX2* gene was not among these SNPs, but *LPL* (rs264) was [27]. LPL catalyses the hydrolysis of triglycerides in plasma triglyceride-rich lipoproteins, chylomicrons and very low density lipoproteins at the capillary endothelial cell surface, providing free fatty acids and glycerol as energy source for tissues [19]. Genetic variation in *LPL* is associated with the levels of circulating LPL activity [5], the plasma concentration of triglycerides [5, 18] and HDL cholesterol [5, 18] and in some [17], albeit not all [18], studies with the risk of CHD. Parenchymal cells in adipose, skeletal and cardiac muscle widely express LPL throughout the body [19]. Cardiac endothelial cells have a particular expression profile including *MEOX2*, a gene that facilitates fatty acid transport into and through heart endothelial cells. As for genetic variation in *LPL* [5, 18], we hypothesised that dysregulation of lipid transport in cardiac endothelial cells might increase CHD risk. Important in this regard is that in our current study, in contrast to the studies on genetic variants of *LPL* [5, 18], we did not find any association of the circulating lipid levels with *MEOX2* variants (data not shown), pointing to a local coronary rather than a systemic underlying mechanism.

Moving to the second hypothetical pathophysiological pathway, MEOX2 is also known as growth arrest-specific homeobox (GAX) [20], During embryonic development, the three muscle lineages express GAX [21]. In adult life, vascular smooth muscle cells also express GAX [21]. Mitogenic stimuli, such as platelet-derived growth factor and angiotensin II or injury of the endothelium [22], inhibit GAX expression, whereas growth arrest signals, such as serum deprivation of cultured cells, enhance its expression and negatively regulate the cell cycle [23]. Observations in transfected cells also point to *MEOX2* as a potentially important regulatory gene inhibiting not only the angiogenic response of endothelial cells to pro-angiogenic factors, but also their response to chronic inflammatory stimulation that normally activates NF-κB [24]. Inflammatory pathways identified in a network analysis of 233 candidate genes play key roles in development of coronary atherosclerosis [27]. These observations [22–24, 27] may offer an alternative explanation why in our current study coronary risk was associated with genetic variation in *MEOX2*. Chen and Gorski did an in silico search for micro-RNA binding sites in the *GAX* 5’UTR and identified consensus sites for multiple candidate micro-RNAs, of which only miR-130a was expressed in proliferating endothelial cells [26]. miR-130a was largely responsible for the down-regulation of GAX expression in response to mitogens and pro-angiogenic factors and antagonised the antiangiogenic activity of GAX [26].

To our knowledge, previously published GWAS results did not demonstrate association between coronary heart disease and *MEOX2*. However, these GWAS studies relied on comparing cases and controls drawn from heterogeneous sources [27–29] or on a retrospective cross-sectional analysis of patients referred for coronary angiography [30]. GWAS case–control studies offer the opportunity for searching for association between CHD and densely distributed SNPs across the whole genome in large numbers of patients and controls. Such studies require significance levels of 10^-6^ to 10^-8^. In contrast, our study was prospective and population-based and tested a prior hypothesis involving only six SNPs in *MEOX2* and four in *TCF15*. We did therefore not rely on such extreme *P*-values, but applied the Benjamin-Hochberg approach for multiple testing. Admittedly, our sample size was smaller than in the GWAS studies. This is particularly relevant for the interaction between *MEOX2* rs4532497 and *TCF15* rs12624577 (Additional file 1: Figure S3), a finding, which although in line with our experimental findings [3] can only be considered as hypothesis generating. Future population-based research projects might address this issue.

We performed the annotation of the genomic context surrounding the SNPs retained for *MEOX2*, based on the data of the ENCODE project (http://www.genome.gov/encode). As shown in Additional file 1: Table S1, rs10777 and rs1050290 map into the 3’UTR and 5’UTR regions, respectively, whereas rs12056299, rs7787043, rs4532497 and rs6959056 are in the first intron of *MEOX2*. Moreover, according to Ensemble 75 annotation (http://www.ensembl.org/Homo_sapiens/Info/Annotation) and GENCODE 22 (http://www.gencodegenes.org/releases/22.html), rs4532497 and rs6959056 also map into the ENSG00000237070 (AC005550.3) antisense non-protein coding gene. In particular, rs4532497 maps into intron 1, whereas rs6959056 maps into exon 4 of ENSG00000237070. rs6959056 and rs1050290 fall into a promoter regulatory region (ENST00000622287) both in human umbilical vein endothelial cells and in human dermal fibroblasts, where MEOX2 is expressed. Further functional studies are needed to investigate the possible modulation of MEOX2 expression. Moreover, rs6959056 in exon 4 of the non-coding transcript ENST00000451240 could affect gene function, although no information on the ENSG00000237070 gene is currently available.

TCF15, also known as Paraxis, is a member of the Twist subfamily of basic helix-loop-helix transcription factors that regulate specification of mesodermal derivatives during vertebrate embryogenesis [31]. TCF15 primes pluripotent cells for differentiation [32]. During dermomyotome formation in *Xenopus laevis*, TCF15 directly activates the expression of MEOX2 [31]. Our experimental studies demonstrated that the MEOX2/TCF15 heterodimer facilitates the transport of fatty acids across cardiac endothelial cells and that in mice haplodeficiency in these genes results in impaired contractility of cardiomyocytes and heart failure [3]. In our population study, we therefore also searched for association between the incidence of well-documented heart failure and variation in the *MEOX2* and *TCF15* genes. Several reasons may explain why such associations were not detected. Indeed, heart failure is a heterogeneous disease caused by a multitude of instigators, including ischaemic or valvular heart disease, comorbidities, or risk factors such as hypertension. Moreover, the diagnosis of heart failure depends on the clinical interpretation of a combination of signs and symptoms, that are difficult to recognise [33, 34].

The present study must be interpreted within the context of some potential limitations. First, even though our analysis was hypothesis-driven based on published evidence from experimental studies [3], we adjusted significance levels for multiple testing according to the number of SNPs tested, using the Benjamini and Hochberg false discovery rate [14]. Even applying the most stringent approach described by Bonferroni did not remove the significance for rs12056299 (*P* = 0.031), rs45324977 (*P* = 0.019) and haplotype *GTCCGC* (*P* = 0.0054) in relation to CHD. On the other hand, we did not consider applying a correction for multiple testing based on the four coronary endpoints. Indeed, such events are highly correlated. Multiple testing is therefore not indicated, because each new test does not provide an independent opportunity for a type-I error [35]. Second, although an observational study cannot prove causation, the Bradford-Hill criteria [36] suggest that the association between coronary risk and genetic variation in *MEOX2* might be causal, taking into account (i) the strength and consistency of the association across different SNPs; (ii) temporality, genetic variability preceding the event; (iii) plausibility based on the experimental studies [3]; and (iv) the analogy observed with genetic variability in *LPL* [5, 17–19]. Third, only few genes regulated by *MEOX2* and TCF15 are currently known. In this regard, it is important to note that we noticed that of the genes overexpressed in cardiac endothelial cells, those most upregulated included genes involved in lipid homeostasis, including *LPL* [3]. Finally, as is common in many population studies, follow-up was inconsistent, with varying numbers of follow-up visits across participants. In addition, participants without blood sample, compared with those included in the analyses (Additional file 1: Table S9), were slightly older (49.4 *vs.* 43.6 years) and had a higher systolic blood pressure (129.5 *vs.* 125.0 mm Hg), resulting in a higher prevalence of hypertension (40.0 *vs.* 24.0 %). We cannot ascertain whether these factors might have biased our analyses.

The clinical implications of our current findings can be gauged by the observation that the attributable and population-attributable CHD risks were similar for the *MEOX2 GTCCGC* carrying state and smoking. Several investigators proposed the use of genetic risk scores based on genome-wide association studies to stratify for the probability of CHD [37, 38]. In the Framingham Heart Study [37], a score consisting of 13 SNPs did not refine the prediction of CHD or cardiovascular disease, but led to modest improvements in risk reclassification. In contrast, in the Rotterdam Study [38], a score based on 152 SNPs was associated with incident CHD, but did not enhance risk prediction. SNP discovery based on prevalent rather than incident CHD might explain these discrepancies [38]. In our current study, genetic variation in *MEOX2* improved IDI and NRI over and beyond the basic model including traditional CHD risk factors.

## Conclusion

Our current study based on a predefined hypothesis generated by data from our experimental studies [3], identified genetic variation in the transcription factor *MEOX2* gene as a novel risk factor for CHD in a white population. However, further experimental studies are required to back-translate our epidemiological observations into underlying molecular mechanisms. Elucidation of these pathways might reveal new targets for the prevention and treatment of CHD.

## Additional file

Additional file 1:
**Table S1.** Common tagging SNPs in *MEOX2.* **Table S2.** *MEOX2* and *TCF15* SNPs and allele and genotype frequencies in unrelated founders. **Table S3.** *MEOX2* and *TCF15* allele and genotype frequencies in 2027 analysed participants. **Table S4.** Sex- and age-standardised CHD rates by *MEOX2* SNPs. **Table S5.** Hazard ratios for CHD by *MEOX2* SNPs in participants free of CHD at baseline. **Table S6.** Sex- and age-standardised CHD rates by *MEOX2* haplotypes. **Table S7.** Hazard ratios for CHD by *MEOX2* haplotypes reconstructed while accounting for pedigree information. **Table S8.** Hazard ratios for CHD by *MEOX2* haplotypes in participants free of CHD at baseline. **Table S9.** Baseline characteristics of participants without blood left for genotyping compared with those included in the analyses. **Figure S1.** Plot of the *MEOX2* gene and flanking regions on chromosome 7. **Figure S2.** Plot of the *TCF15* gene and flanking regions on chromosome 20. **Figure S3.** Interaction between *TCF15* rs12624577 and *MEOX2* rs4532497. **Figure S4.** Incidence of coronary endpoints, myocardial infarction and coronary revascularisation in *MEOX2 GTCCGC* carriers and non-carriers.

## Abbreviations

CHD: Coronary heart disease
CD36: Cluster of differentiation 36
DNA: Deoxyribonucleic acid
ENCODE: Encyclopaedia of DNA elements
GAX: Growth arrest-specific homeobox
GENCODE: Encyclopaedia of genes and genes variants
FLEMENGHO: Flemish Study on Environment, Genes and Health Outcomes
GWAS: Genome-wide association study
HDL: High density lipoprotein
IDI: Integrated discrimination improvement
LPL: Lipoprotein lipase
MEOX2: Mesenchyme homeobox 2
miRNA: Micro Ribonucleic acid
NF-κB: Nuclear factor of kappa light polypeptide gene enhancer in B cells
NRI: Net reclassification improvement
SNP: Single nucleotide polymorphism
TCF15: Transcription factor 15
UTR: Untranslated regions
