# Supplementary material for: Coronary risk in relation to genetic variation in MEOX2 and TCF15 in a Flemish population
Source: BMC Genet. 2015 Oct 1;16:116. doi: 10.1186/s12863-015-0272-2 (PMC4591634; doi:10.1186/s12863-015-0272-2)
Supplement: Additional file 1: — Table S1. Common tagging SNPs in MEOX2. Table S2. MEOX2 and TCF15 SNPs and allele and genotype frequencies in unrelated founders. Table S3. MEOX2 and TCF15 allele and genotype frequencies in 2027 analysed participants. Table S4. Sex- and age-standardised CHD rates by MEOX2 SNPs. Table S5. Hazard ratios for CHD by MEOX2 SNPs in participants free of CHD at baseline. Table S6. Sex- and age-standardised CHD rates by MEOX2 haplotypes. Table S7. Hazard ratios for CHD by MEOX2 haplotypes reconstructed while accounting for pedigree information. Table S8. Hazard ratios for CHD by MEOX2 haplotypes in participants free of CHD at baseline. Table S9. Baseline characteristics of participants without blood left for genotyping compared with those included in the analyses. Figure S1. Plot of the MEOX2 gene and flanking regions on chromosome 7. Figure S2. Plot of the TCF15 gene and flanking regions on chromosome 20. Figure S3. Interaction between TCF15 rs12624577 and MEOX2 rs4532497. Figure S4. Incidence of coronary endpoints, myocardial infarction and coronary revascularisation in MEOX2 GTCCGC carriers and non-carriers. [file 12863_2015_272_MOESM1_ESM.doc]

**BMC Genetics**

**Additional file 1**

This web appendix formed part of the original submission and has been peer reviewed.
Supplement to: *Coronary risk in relation to genetic variation in MEOX2 and TCF15 in a Flemish population*.

**Table of contents**

**Table S1** Common tagging SNPs in *MEOX2* page **2**

**Table S2** *MEOX2* and *TCF15* SNPs and allele and genotype frequencies in unrelated founders page **3**

**Table S3** *MEOX2* and *TCF15* allele and genotype frequencies in ~~in~~ 2027 analysed participants page **4**

**Table S4** Sex- and age-standardised CHD rates by *MEOX2* SNPs page **5**

**Table S5** Hazard ratios for CHD by *MEOX2* SNPs in participants free of
 CHD at baseline page **7**

**Table S6** Sex- and age-standardised CHD rates by *MEOX2* haplotypes page **8**

**Table S7** Hazard ratios for CHD by *MEOX2* haplotypes reconstructed while
 accounting for pedigree information page **9**

**Table S8** Hazard ratios for CHD by *MEOX2* haplotypes in participants free of
 CHD at baseline page **10**

**Table S9** Baseline characteristics of participants without blood left for genotyping
 compared with those included in the analyses page **11**

**Figure S1** Plot of the *MEOX2* gene and flanking regions on chromosome 7 page **12**

**Figure S2** Plot of the *TCF15* gene and flanking regions on chromosome 20 page **13**

**Figure S3** Interaction between *TCF15* rs12624577 and *MEOX2* rs4532497 page **14**

**Figure S4** Incidence of coronary endpoints, myocardial infarction and coronary
 revascularisation in *MEOX2* *GTCCGC* carriers and non-carriers page **15**

**Table S1**

**Tagging SNPs in *MEOX2* that are in linkage disequilibrium with 92 tagged SNPs**

| SNP | Position  (base pairs) | Location | Call rate | N° tagged SNPs | Tagged SNPs |
| --- | --- | --- | --- | --- | --- |
| rs6946099 | 15648861 | 3’downstream | 0.97 | 7 | rs57830137, rs17168897, rs73071878, rs12699747, rs55653754, rs6976202 |
| rs10777 | 15651188 | 3’UTR | 1.00 | 6 | rs17168900, rs17199, rs2237492, rs4141308, rs2237494, rs7783223 |
| rs7800473 | 15653054 | intron | 0.91 | 30 | rs9638727, rs74476414, rs114077391, rs10259769, rs13227097, rs6955921, rs6949297, rs6969519, rs6950541, rs6970492, rs73298592, rs6461196, rs10275641, rs10246570, rs7788980, rs73298595, rs118111792, rs7793128, rs17168904, rs7793310, rs7812247, rs7793334, rs7793459, rs7793498, rs6960768, rs17168905, rs6943140, rs6961370, rs17168906, rs10266294 |
| rs13438001 | 15680072 | intron | 0.95 | 15 | rs76902068, rs2282935, rs34460507, rs62439049, rs76054184, rs3801421, rs10486770, rs10228856, rs10486771, rs13240689, rs11982768, rs10249754, rs2389538, rs34751158, rs6461200 |
| rs12056299 | 15696151 | intron | 0.98 | 2 | rs917437, rs7786884 |
| rs7787043 | 15698178 | intron | 0.98 | 8 | rs62439053, rs981597, rs10282109, rs6969225, rs6947658, rs6972599, rs6978527, rs12533130 |
| rs758297 | 15707445 | intron | 0.97 | 4 | rs12533863, rs34994232, rs57483785, rs73288145 |
| rs4532497 | 15712852 | intron | 0.99 | 0 |  |
| rs10263561 | 15713039 | intron | 0.95 | 11 | rs10046550, rs10046551, rs10263831, rs6461202, rs6980299, rs957104, rs28397811, rs4380818, rs28739453, rs11767931, rs11768065 |
| rs6959056 | 15721466 | intron | 0.98 | 5 | rs73060529, rs16878585, rs12540302, rs3801429, rs3801430 |
| rs1050290 | 15726066 | 5’UTR | 0.99 | 2 | rs11975534, rs917441 |
| rs740566 | 15727202 | 5’upstream | 0.97 | 2 | rs740567, rs3807879 |

SNP ID is a GenBank ID number (National Center for Biotechnology Information, Bethesda, MD). Position and location were taken from the most recent human genome sequence assemblies (NCBI Build 37.3). SNPs with call rate ≥0.98 were included for final analysis. Tagged SNPs have *r2* ≥ 0.80 with the *MEOX2* markers.

**Table S2**

**Allele and genotype frequencies in 825 unrelated founders**

| **Single nucleotide polymorphisms by gene** | | | | | |  | **Allele and genotype frequencies** | | | | | | | |
| --- | --- | --- | --- | --- | --- | --- | --- | --- | --- | --- | --- | --- | --- | --- |
| Gene | SNP | Position  (base pairs) | Base pairs | Location | *r^2^* |  | *A1* (%) | *A2* (%) |  | *G1* (%) | *G2* (%) | *G3* (%) |  | *P* |
| *MEOX2* | rs10777 | 7:15651158 | 0 | 3’UTR | … |  | *G* (27.9) | *T* (72.1) |  | *GG* (8.2) | *TG* (39.3) | *TT* (52.5) |  | 0.50 |
| *MEOX2* | rs12056299 | 7:15696151 | 44993 | intron | 0.43 |  | *T* (21.5) | *C* (78.5) |  | *TT* (5.2) | *CT* (32.6) | *CC* (62.2) |  | 0.32 |
| *MEOX2* | rs7787043 | 7:15698178 | 47020 | intron | 0.31 |  | *C* (33.5) | *T* (66.5) |  | *CC* (11.8) | *TC* (43.5) | *TT* (44.7) |  | 0.50 |
| *MEOX2* | rs4532497 | 7:15712852 | 61694 | intron | 0.28 |  | *C* (29.3) | *T* (70.7) |  | *CC* (9.1) | *TC* (40.3) | *TT* (50.6) |  | 0.46 |
| *MEOX2* | rs6959056 | 7:15721466 | 70308 | intron | 0.02 |  | *A* (43.0) | *G* (57.0) |  | *AA* (17.6) | *GA* (50.8) | *GG* (31.6) |  | 0.30 |
| *MEOX2* | rs1050290 | 7:15726066 | 74908 | 5’UTR | 0.21 |  | *C* (36.4) | *T* (63.6) |  | *CC* (14.2) | *TC* (44.5) | *TT* (41.3) |  | 0.30 |
| *TCF15* | rs6116745 | 20:583587 | 0 | 3’UTR | … |  | *A* (37.2) | *G* (62.8) |  | *AA* (13.3) | *GA* (47.7) | *GG* (39.0) |  | 0.56 |
| *TCF15* | rs282162 | 20:586955 | 3368 | Intron | 0.01 |  | *C* (37.4) | *A* (62.6) |  | *CC* (11.8) | *AC* (46.8) | *AA* (41.4) |  | 0.73 |
| *TCF15* | rs3761308 | 20:591694 | 8107 | 5’UTR | 0.02 |  | *T* (10.0) | *C* (90.0) |  | *TT* (0.7) | *CT* (18.6) | *CC* (80.7) |  | 0.38 |
| *TCF15* | rs12624577 | 20:611176 | 8233 | 5’UTR | 0.001 |  | *C* (39.0) | *T* (61.0) |  | *CC* (15.4) | *TC* (47.2) | *TT* (37.4) |  | 0.80 |

*A1* and *A2* refer to the minor and major alleles; *G1*, *G2* and *G3* indicate homozygotes and heterozygotes of the minor allele and homozygotes of the major allele, respectively. Base pairs and *r2* are measures for the distance from and the linkage disequilibrium with rs10777 in *MEOX2* and rs61166745 in *TCF15*. *P*-values are for departure from Hardy–Weinberg equilibrium.

**Table S3**

**Allele and genotype frequencies in 2027 analysed participants**

| **SNP by gene** |  | **Base pairs** |  | **Allele frequencies** | |  | **Genotype frequencies** | | |
| --- | --- | --- | --- | --- | --- | --- | --- | --- | --- |
|  |  | ***A1* / *A2*** |  | ***A1*** | ***A2*** |  | ***A1A1*** | ***A2A1*** | ***A2A2*** |
| ***MEOX2*** |  |  |  |  |  |  |  |  |  |
| rs10777 |  | *G / T* |  | 1056 (26.0) | 2998 (74.0) |  | 145 (7.1) | 766 (37.8) | 1116 (55.1) |
| rs12056299 |  | *T / C* |  | 854 (21.1) | 3200 (78.9) |  | 90 (4.4) | 674 (33.3) | 1263 (62.3) |
| rs7787043 |  | *C / T* |  | 1337 (33.0) | 2717 (67.0) |  | 226 (11.2) | 885 (43.7) | 916 (45.2) |
| rs4532497 |  | *C / T* |  | 1175 (29.0) | 2879 (71.0) |  | 171 (8.4) | 833 (41.1) | 1023 (50.5) |
| rs6959056 |  | *A / G* |  | 1743 (43.0) | 2311 (57.0) |  | 361 (17.8) | 1021 (50.4) | 645 (31.8) |
| rs1050290 |  | *C / T* |  | 1465 (36.1) | 2589 (63.9) |  | 271 (13.4) | 923 (45.5) | 833 (41.1) |
| ***TCF15*** |  |  |  |  |  |  |  |  |  |
| *rs6116745* |  | *A / G* |  | 1562 (38.5) | 2492 (61.5) |  | 322 (15.9) | 918 (45.3) | 787 (38.8) |
| *rs282162* |  | *C / A* |  | 1427 (35.2) | 2627 (64.8) |  | 239 (11.8) | 949 (46.8) | 839 (41.4) |
| *rs3761308* |  | *T / C* |  | 391 (9.6) | 3663 (90.4) |  | 15 (0.7) | 361 (17.8) | 1651 (81.5) |
| rs12624577 |  | *C* / *T* |  | 1559 (38.5) | 2495 (61.5) |  | 295 (14.6) | 969 (47.8) | 763 (37.6) |

*A1* and *A2* refer to the minor and major alleles; *A1A1*, *A2A1* and *A2A2* indicate homozygotes and heterozygotes of the minor allele and homozygotes of the major allele, respectively. Values are number of alleles or genotypes (%).

**Table S4**

**Sex- and age-standardised CHD rates by *MEOX2* SNPs (starts)**

| **SNP** Event |  | **Minor allele**  **carriers** | |  | **Major allele**  **homozygotes** | |  | ***P*** | ***P***BH |
| --- | --- | --- | --- | --- | --- | --- | --- | --- | --- |
|  |  | *n* | Rate |  | *n* | Rate |  |  |  |
| **rs10777** |  | 911 | ***GG+TG*** |  | 1116 | ***TT*** |  |  |  |
| All coronary events |  | 59 | 4.54 (3.38–5.60) |  | 47 | 2.94 (2.10–3.79) |  | 0.029 | 0.034 |
| Myocardial infarction |  | 31 | 2.27 (1.47–3.07) |  | 22 | 1.34 (0.78–1.90) |  | 0.062 | 0.074 |
| Coronary revascularisation |  | 42 | 3.21 (2.24–4.19) |  | 36 | 2.24 (1.51–2.98) |  | 0.12 | 0.14 |
| Ischaemic cardiomyopathy |  | 16 | 1.19 (0.61–1.77) |  | 6 | 0.35 (0.07–0.64) |  | 0.012 | 0.047 |
| **rs12056299** |  | 764 | ***TT+CT*** |  | 1263 | ***CC*** |  |  |  |
| All coronary events |  | 51 | 4.90 (3.56–6.24) |  | 55 | 2.97 (2.19–3.75) |  | 0.015 | 0.023 |
| Myocardial infarction |  | 28 | 2.54 (1.60–3.49) |  | 25 | 1.31 (0.80–1.82) |  | 0.024 | 0.048 |
| Coronary revascularisation |  | 37 | 3.52 (2.38–4.65) |  | 41 | 2.20 (1.52–2.87) |  | 0.050 | 0.075 |
| Ischaemic cardiomyopathy |  | 14 | 1.34 (0.64–2.04) |  | 8 | 0.41 (0.13–0.69) |  | 0.016 | 0.047 |
| **rs7787043** |  | 1111 | ***CC+TC*** |  | 916 | ***TT*** |  |  |  |
| All coronary events |  | 74 | 4.62 (3.57–5.67) |  | 32 | 2.39 (1.56–3.23) |  | 0.0011 | 0.0066 |
| Myocardial infarction |  | 38 | 2.25 (1.54–2.97) |  | 15 | 1.09 (0.54–1.65) |  | 0.012 | 0.048 |
| Coronary revascularisation |  | 54 | 3.34 (2.45–4.23) |  | 24 | 1.79 (1.07–2.51) |  | 0.0078 | 0.029 |
| Ischaemic cardiomyopathy |  | 16 | 0.95 (0.48–1.41) |  | 6 | 0.43 (0.09–0.78) |  | 0.083 | 0.17 |

**Table S4**

**Sex- and age-standardised CHD rates by *MEOX2* SNPs (continued)**

| **SNP** Event |  | **Minor allele**  **carriers** | |  | **Major allele**  **homozygotes** | | | |  | | ***P*** | | ***P***BH | |
| --- | --- | --- | --- | --- | --- | --- | --- | --- | --- | --- | --- | --- | --- | --- |
|  |  | *n* | Rate |  | *n* | | Rate | |  | |  |  |  |  |
| **rs4532497** |  | 1004 | ***CC+TC*** |  | | 1023 | | ***TT*** | |  | |  | |  |
| All coronary events |  | 66 | 4.62 (3.51–5.73) |  | | 40 | | 2.71 (1.86–3.55) | |  | | 0.0073 | | 0.022 |
| Myocardial infarction |  | 33 | 2.23 (1.47–2.99) |  | | 20 | | 1.31 (0.73–1.88) | |  | | 0.058 | | 0.074 |
| Coronary revascularisation |  | 50 | 3.45 (2.50–4.41) |  | | 28 | | 1.89 (1.18–2.59) | |  | | 0.0096 | | 0.029 |
| Ischaemic cardiomyopathy |  | 14 | 0.94 (0.45–1.44) |  | | 8 | | 0.52 (0.16–0.88) | |  | | 0.17 | | 0.26 |
| **rs6959056** |  | 1382 | ***AA+GA*** |  | | 645 | | ***GG*** | |  | |  | |  |
| All coronary events |  | 59 | 3.01 (2.24–3.79) |  | | 47 | | 5.04 (3.60–6.48) | |  | | 0.015 | | 0.023 |
| Myocardial infarction |  | 27 | 1.31 (0.82–1.81) |  | | 26 | | 2.69 (1.66–3.72) | |  | | 0.019 | | 0.048 |
| Coronary revascularisation |  | 46 | 2.34 (1.66–3.03) |  | | 32 | | 3.37 (2.20–4.53) | |  | | 0.14 | | 0.14 |
| Ischaemic cardiomyopathy |  | 12 | 0.60 (0.26–0.95) |  | | 10 | | 1.01 (0.39–1.64) | |  | | 0.26 | | 0.26 |
| **rs1050290** |  | 1194 | ***CC+TC*** |  | | 833 | | ***TT*** | |  | |  | |  |
| All coronary events |  | 73 | 4.22 (3.26–5.19) |  | | 33 | | 2.76 (1.80–3.71) | |  | | 0.034 | | 0.034 |
| Myocardial infarction |  | 36 | 2.01 (1.35–2.66) |  | | 17 | | 1.39 (0.72–2.05) | |  | | 0.19 | | 0.19 |
| Coronary revascularisation |  | 55 | 3.16 (2.33–3.99) |  | | 23 | | 1.90 (1.12–2.69) | |  | | 0.032 | | 0.064 |
| Ischaemic cardiomyopathy |  | 15 | 0.87 (0.44–1.30) |  | | 6 | | 0.51 (0.10–0.92) | |  | | 0.23 | | 0.26 |

Rates expressed per 1000 person-years were standardised for sex and age group (<40, 40–59, ≥60 years) by the direct method. *P* and *P*BH indicate the significance of the rate differences without and with Benjamini-Hochberg’s correction for multiple testing.

**Table S5**

**Multivariable-adjusted hazard ratios for CHD by *MEOX2* SNPs in 1986 participants free of CHD at baseline**

| **SNP**  Event |  | **N° events/at risk** | |  | **Hazard ratio** |  | ***P*** | ***P***BH |
| --- | --- | --- | --- | --- | --- | --- | --- | --- |
|  |  | ***Minor allele carriers*** | ***Major allele homozygotes*** |  |  |  |  |  |
| **rs10777** |  | ***GG+TG*** | ***TT*** |  |  |  |  |  |
| All coronary events |  | 54/889 | 41/1097 |  | 1.73 (1.15–2.60) |  | 0.0090 | 0.014 |
| Myocardial infarction |  | 29 | 19 |  | 1.97 (1.10–3.52) |  | 0.022 | 0.026 |
| Coronary revascularisation |  | 39 | 32 |  | 1.55 (0.96–2.50) |  | 0.070 | 0.084 |
| Ischaemic cardiomyopathy |  | 11 | 5 |  | 4.57 (1.61–12.96) |  | 0.0043 | 0.017 |
| **rs12056299** |  | ***TT+CT*** | ***CC*** |  |  |  |  |  |
| All coronary events |  | 47/746 | 48/1240 |  | 1.88 (1.28–2.78) |  | 0.0014 | 0.0042 |
| Myocardial infarction |  | 26 | 22 |  | 2.27 (1.28–4.02) |  | 0.0052 | 0.031 |
| Coronary revascularisation |  | 35 | 36 |  | 1.81 (1.13–2.90) |  | 0.014 | 0.041 |
| Ischaemic cardiomyopathy |  | 10 | 6 |  | 3.47 (1.44–8.36) |  | 0.0057 | 0.017 |
| **rs7787043** |  | ***CC+TC*** | ***TT*** |  |  |  |  |  |
| All coronary events |  | 67/1084 | 28/902 |  | 1.88 (1.21–2.92) |  | 0.0053 | 0.011 |
| Myocardial infarction |  | 36 | 12 |  | 2.27 (1.21–4.24) |  | 0.010 | 0.031 |
| Coronary revascularisation |  | 49 | 22 |  | 1.75 (1.03–2.97) |  | 0.039 | 0.078 |
| Ischaemic cardiomyopathy |  | 12 | 4 |  | 2.82 (0.90–8.79) |  | 0.074 | 0.089 |
| **rs4532497** |  | ***CC+TC*** | ***TT*** |  |  |  |  |  |
| All coronary events |  | 60/981 | 35/1005 |  | 1.94 (1.30–2.91) |  | 0.0013 | 0.0042 |
| Myocardial infarction |  | 31 | 17 |  | 2.15 (1.13–4.10) |  | 0.020 | 0.026 |
| Coronary revascularisation |  | 45 | 26 |  | 1.89 (1.19–3.00) |  | 0.0074 | 0.041 |
| Ischaemic cardiomyopathy |  | 11 | 5 |  | 2.72 (0.94–7.88) |  | 0.064 | 0.089 |
| **rs6959056** |  | ***AA+GA*** | ***GG*** |  |  |  |  |  |
| All coronary events |  | 56/1360 | 39/626 |  | 0.67 (0.44–1.00) |  | 0.049 | 0.049 |
| Myocardial infarction |  | 26 | 22 |  | 0.53 (0.31–0.91) |  | 0.021 | 0.026 |
| Coronary revascularisation |  | 44 | 27 |  | 0.76 (0.47–1.23) |  | 0.26 | 0.26 |
| Ischaemic cardiomyopathy |  | 9 | 7 |  | 0.79 (0.32–1.99) |  | 0.62 | 0.62 |
| **rs1050290** |  | ***CC+TC*** | ***TT*** |  |  |  |  |  |
| All coronary events |  | 67/1169 | 28/817 |  | 1.64 (1.07–2.51) |  | 0.023 | 0.027 |
| Myocardial infarction |  | 34 | 14 |  | 1.71 (0.89–3.29) |  | 0.11 | 0.011 |
| Coronary revascularisation |  | 50 | 21 |  | 1.60 (0.98–2.62) |  | 0.060 | 0.084 |
| Ischaemic cardiomyopathy |  | 13 | 3 |  | 3.56 (1.00–12.68) |  | 0.051 | 0.089 |

Numbers of events do not add up, because only the first event in each category was analysed. Hazard ratios (95% confidence interval) express the risk of minor allele carriers *vs.* major allele homozygotes, account for family clusters, and were adjusted for baseline characteristics including sex, age, body mass index, systolic pressure, total-to-HDL cholesterol ratio, smoking and drinking, and antihypertensive drug treatment. *P* and *P*BH indicate the significance of the hazard ratios without and with Benjamini-Hochberg’s correction for multiple testing.

**Table S6**

**Sex- and age-standardised CHD rates by *MEOX2* haplotypes**

| **Haplotype**  Event |  | **Carriers** | |  |  | **Non-carriers** |  | ***P*** | ***P***BH |
| --- | --- | --- | --- | --- | --- | --- | --- | --- | --- |
|  |  | *n* | Rate |  | *n* | Rate |  |  |  |
| ***TCTTAT*** |  | 951 |  |  | 1076 |  |  |  |  |
| All coronary events |  | 40 | 3.01 (2.07–3.95) |  | 66 | 4.21 (3.20–5.22) |  | 0.089 | 0.13 |
| Myocardial infarction |  | 20 | 1.45 (0.81–2.08) |  | 33 | 2.00 (1.32–2.68) |  | 0.25 | 0.37 |
| Coronary revascularisation |  | 30 | 2.26 (1.45–3.08) |  | 48 | 3.03 (2.18–3.89) |  | 0.20 | 0.20 |
| Ischaemic cardiomyopathy |  | 6 | 0.47 (0.09–0.84) |  | 16 | 0.96 (0.49–1.44) |  | 0.11 | 0.16 |
| ***TCTTGT*** |  | 937 |  |  | 1090 |  |  |  |  |
| All coronary events |  | 46 | 3.48 (2.47–4.49) |  | 60 | 3.81 (2.85–4.77) |  | 0.64 | 0.64 |
| Myocardial infarction |  | 26 | 1.90 (1.17–2.63) |  | 27 | 1.65 (1.03–2.27) |  | 0.61 | 0.61 |
| Coronary revascularisation |  | 30 | 2.24 (1.43–3.04) |  | 48 | 3.03 (2.17–3.88) |  | 0.19 | 0.20 |
| Ischaemic cardiomyopathy |  | 9 | 0.65 (0.22–1.07) |  | 13 | 0.82 (0.38–1.27) |  | 0.57 | 0.57 |
| ***GTCCGC*** |  | 614 |  |  | 1413 |  |  |  |  |
| All coronary events |  | 43 | 5.26 (3.69–6.84) |  | 63 | 3.03 (2.28–3.78) |  | 0.012 | 0.036 |
| Myocardial infarction |  | 23 | 2.64 (1.56–3.72) |  | 30 | 1.41 (0.9–1.91) |  | 0.042 | 0.13 |
| Coronary revascularisation |  | 33 | 4.03 (2.65–5.4) |  | 45 | 2.14 (1.51–2.77) |  | 0.014 | 0.043 |
| Ischaemic cardiomyopathy |  | 11 | 1.3 (0.53–2.06) |  | 11 | 0.51 (0.21–0.81) |  | 0.060 | 0.16 |

Rates expressed per 1000 person-years were standardised for sex and age group (<40, 40–59, ≥60 years) by the direct method. Letters coding the haplotypes refer to the rs10777, rs12056299, rs7787043, rs4532497, rs6959056 and rs1050290 alleles. Haplotypes were reconstructed using the expectation-maximisation algorithm as implemented in the PROC HAPLOTYPE procedure of the SAS software version 9.3. Haplotypes with a frequency of ≥10% were carried forward in the analysis. *P* and *P*BH indicate the significance of the rate differences without and with Benjamini-Hochberg’s correction for multiple testing.

**Table S7**

**Multivariable-adjusted hazard ratios for CHD by *MEOX2* haplotypes estimated by taking into account pedigrees**

| **Haplotypes**  Event |  | **N° events/at risk** | |  | **Hazard ratio** |  | ***P*** | ***P***BH |
| --- | --- | --- | --- | --- | --- | --- | --- | --- |
|  |  | ***Carrier*** | ***Non-carrier*** |  |  |  |  |  |
| ***TCTTAT*** |  |  |  |  |  |  |  |  |
| All coronary events |  | 39/944 | 67/1083 |  | 0.73 (0.48–1.10) |  | 0.13 | 0.20 |
| Myocardial infarction |  | 20 | 33 |  | 0.81 (0.44–1.47) |  | 0.48 | 0.52 |
| Coronary revascularisation |  | 29 | 49 |  | 0.74 (0.46–1.18) |  | 0.20 | 0.20 |
| Ischaemic cardiomyopathy |  | 6 | 16 |  | 0.59 (0.22–1.57) |  | 0.29 | 0.29 |
| ***TCTTGT*** |  |  |  |  |  |  |  |  |
| All coronary events |  | 45/915 | 61/1112 |  | 0.88 (0.57–1.37) |  | 0.57 | 0.57 |
| Myocardial infarction |  | 26 | 27 |  | 1.21 (0.68–2.17) |  | 0.52 | 0.52 |
| Coronary revascularisation |  | 29 | 49 |  | 0.72 (0.44–1.16) |  | 0.18 | 0.18 |
| Ischaemic cardiomyopathy |  | 9 | 13 |  | 0.59 (0.24–1.44) |  | 0.25 | 0.29 |
| ***GTCCGC*** |  |  |  |  |  |  |  |  |
| All coronary events |  | 43/616 | 63/1411 |  | 1.78 (1.24–2.55) |  | 0.0019 | 0.0057 |
| Myocardial infarction |  | 23 | 30 |  | 1.96 (1.16–3.31) |  | 0.012 | 0.036 |
| Coronary revascularisation |  | 33 | 45 |  | 1.87 (1.20–2.90) |  | 0.0058 | 0.017 |
| Ischaemic cardiomyopathy |  | 11 | 11 |  | 3.16 (1.41–7.08) |  | 0.0053 | 0.016 |

Numbers of events do not add up, because only the first event in each category was analysed. Letters coding the haplotypes refer to the rs10777, rs12056299, rs7787043, rs4532497, rs6959056 and rs1050290 alleles (see *Additional file 1: Tables S1* and *S2*). Haplotypes were inferred by taking into account pedigree information using SHAPEIT version 2. Three haplotypes, *TCTTAT* (27.2%), *TCTTAT* (25.7%), and *GTCCGC* (16.6%) with a frequency over 10% were carried through in the analysis. Hazard ratios (95% confidence interval) express the risk associated with carrying *vs.* not carrying a haplotype, account for family clusters, and were adjusted for baseline characteristics including sex, age, body mass index, systolic pressure, total-to-HDL cholesterol ratio, smoking and drinking, and antihypertensive drug treatment. *P* and *P*BH indicate the significance of the hazard ratios without and with Benjamini-Hochberg’s correction for multiple testing.

**Table S8**

**Multivariable-adjusted hazard ratios for CHD by *MEOX2* haplotypes in 1986 participants free of CHD at baseline**

| **Haplotypes**  Event |  | **N° events/at risk** | |  | **Hazard ratio** |  | ***P*** | ***P***BH |
| --- | --- | --- | --- | --- | --- | --- | --- | --- |
|  |  | ***Carrier*** | ***Non-carrier*** |  |  |  |  |  |
| ***TCTTAT*** |  |  |  |  |  |  |  |  |
| All coronary events |  | 38/935 | 57/1051 |  | 0.79 (0.51–1.21) |  | 0.28 | 0.30 |
| Myocardial infarction |  | 19 | 29 |  | 0.80 (0.44–1.46) |  | 0.47 | 0.70 |
| Coronary revascularisation |  | 29 | 42 |  | 0.81 (0.50–1.32) |  | 0.40 | 0.40 |
| Ischaemic cardiomyopathy |  | 4 | 12 |  | 0.47 (0.15–1.51) |  | 0.20 | 0.20 |
| ***TCTTGT*** |  |  |  |  |  |  |  |  |
| All coronary events |  | 38/918 | 57/1068 |  | 0.79 (0.51–1.23) |  | 0.30 | 0.30 |
| Myocardial infarction |  | 21 | 27 |  | 0.98 (0.53–1.82) |  | 0.94 | 0.94 |
| Coronary revascularisation |  | 26 | 45 |  | 0.69 (0.42–1.14) |  | 0.15 | 0.22 |
| Ischaemic cardiomyopathy |  | 6 | 10 |  | 0.50 (0.18–1.39) |  | 0.18 | 0.20 |
| ***GTCCGC*** |  |  |  |  |  |  |  |  |
| All coronary events |  | 40/600 | 55/1386 |  | 1.95 (1.33–2.85) |  | 0.0006 | 0.0018 |
| Myocardial infarction |  | 22 | 26 |  | 2.21 (1.16–3.85) |  | 0.0055 | 0.017 |
| Coronary revascularisation |  | 31 | 40 |  | 2.03 (1.27–3.23) |  | 0.0030 | 0.0090 |
| Ischaemic cardiomyopathy |  | 8 | 8 |  | 3.54 (1.42–8.83) |  | 0.0067 | 0.020 |

Numbers of events do not add up, because only the first event in each category was analysed. Letters coding the haplotypes refer to the rs10777, rs12056299, rs7787043, rs4532497, rs6959056 and rs1050290 alleles (see *Additional file 1: Tables S1* and *S2*). Haplotypes were reconstructed using the expectation-maximisation algorithm as implemented in the PROC HAPLOTYPE procedure of the SAS software version 9.3. Hazard ratios (95% confidence interval) express the risk associated with carrying *vs.* not carrying a haplotype, account for family clusters, and were adjusted for baseline characteristics including sex, age, body mass index, systolic pressure, total-to-HDL cholesterol ratio, smoking and drinking, and antihypertensive drug treatment. *P* and *P*BH indicate the significance of the hazard ratios without and with Benjamini-Hochberg’s correction for multiple testing.

**Table S9**

**Baseline characteristics of participants without blood left for genotyping compared with those included in the analyses**

| **Characteristic** | **Participants without blood sample** | **Analysed** **participants** | **P** |
| --- | --- | --- | --- |
| N° | 521 | 2027 |  |
| N° with characteristics (%) |  |  |  |
| Women | 257 (49.3) | 1034 (51.0) | 0.49 |
| Current smoker | 138 (26.5) | 605 (29.9) | 0.11 |
| Drinking alcohol | 138 (26.5) | 586 (28.9) | 0.29 |
| Diabetes mellitus | 14 (2.6) | 33 (1.6) | 0.074 |
| Hypertension | 203 (40.0) | 486 (24.0) | <0.0001 |
| Mean of characteristic (±SD) |  |  |  |
| Age, years | 49.4±17.3 | 43.6±14.3 | <0.0001 |
| Body mass index, kg/m^2^ | 25.8±4.5 | 25.7±4.3 | 0.45 |
| Waist-to-hip ratio | 0.86±0.10 | 0.85±0.09 | 0.10 |
| Systolic blood pressure, mm Hg | 129.5±19.8 | 125.0±15.4 | <0.0001 |
| Diastolic blood pressure, mm Hg | 77.1±10.9 | 76.2±9.5 | 0.040 |
| Total cholesterol, mmol/L | 5.64±1.26 | 5.49±1.15 | 0.010 |
| HDL cholesterol, mmol/L | 1.34±0.36 | 1.37±0.39 | 0.18 |
| Total-to-HDL cholesterol ratio | 4.43±1.56 | 4.29±1.66 | 0.19 |

HDL cholesterol refers to the serum concentration of high-density lipoprotein cholesterol. Diabetes mellitus was a fasting or random plasma glucose level exceeding 7.0 or 11.1 mmol/L, or use of antidiabetic agents. Hypertension was a blood pressure of ≥140 mm Hg systolic or ≥90 mm Hg diastolic or use of antihypertensive drugs.


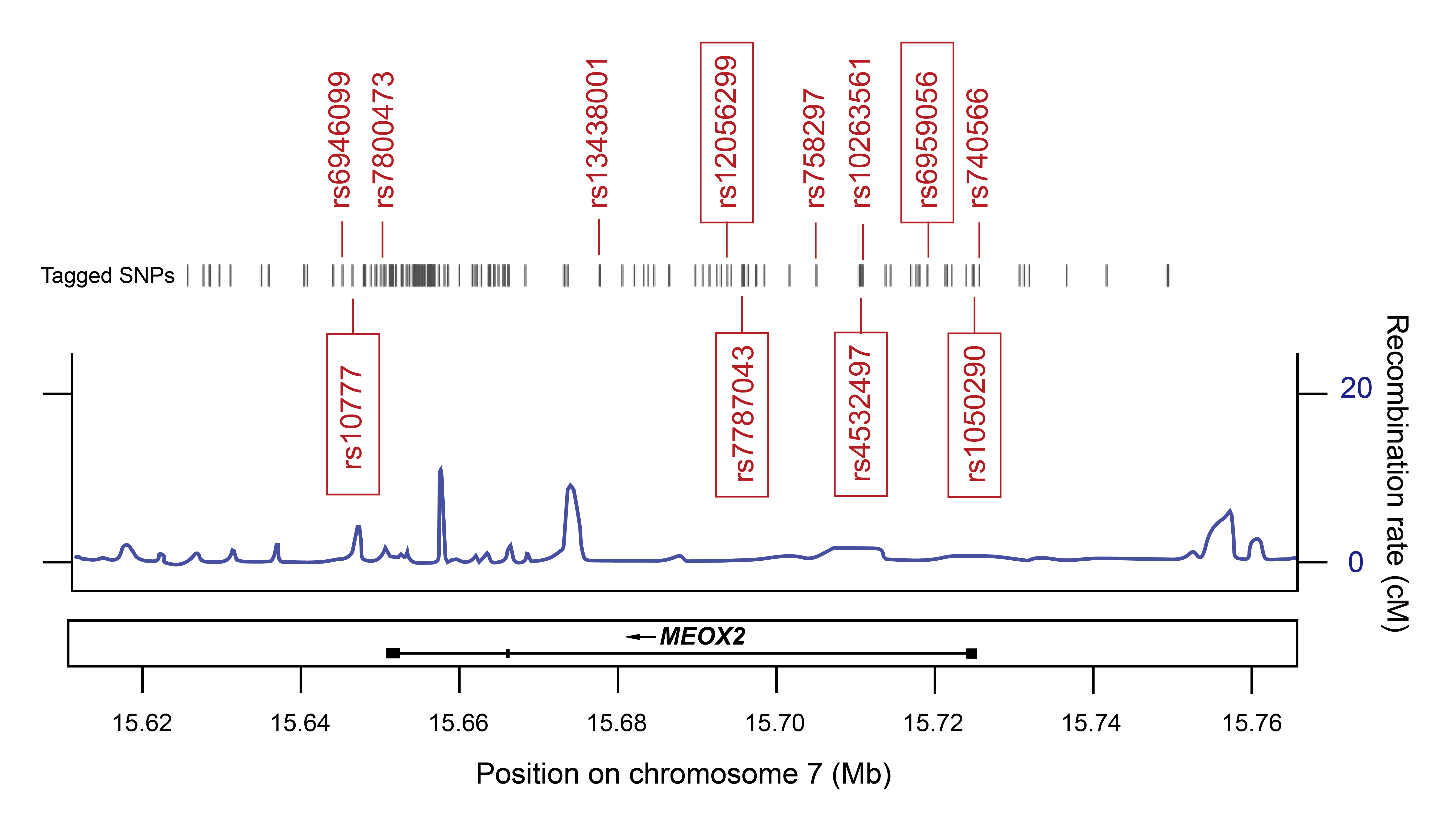


**Figure S1**

Plot of the *MEOX2* gene and flanking regions on chromosome 7 (p22.1–21.3). The x‑axis represents the physical position on the chromosome (build 37, hg19). The y-axis and the line indicate the recombination rate. The selected 12 SNPs (rs number and position given) are in linkage disequilibrium (*r^2^* ≥ 0.80) with tagged SNPs denoted by vertical lines. The six framed SNPs with call rate ≥0.98 were retained in the analysis. More information is available in *Additional file 1: Tables S1*.


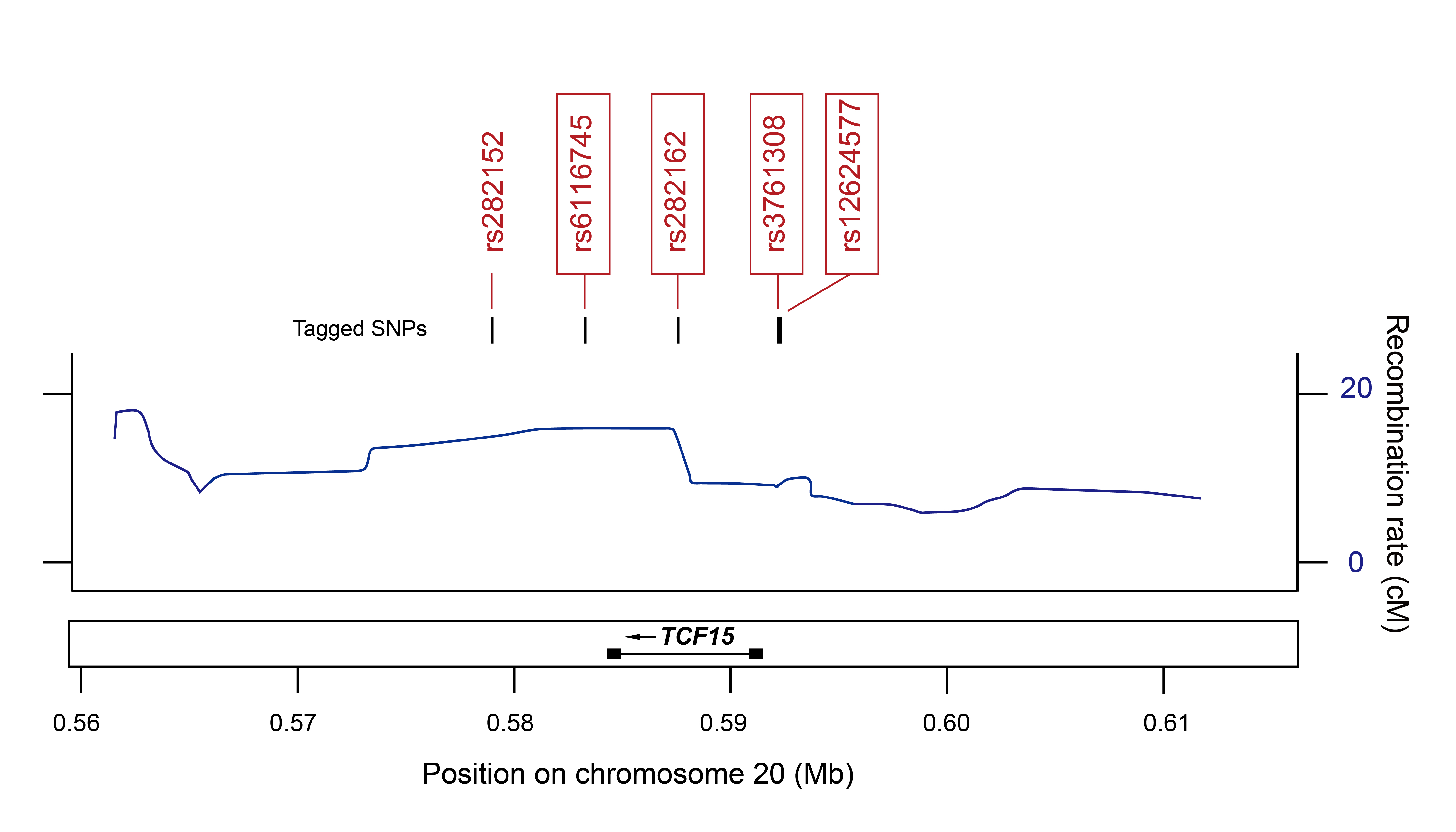


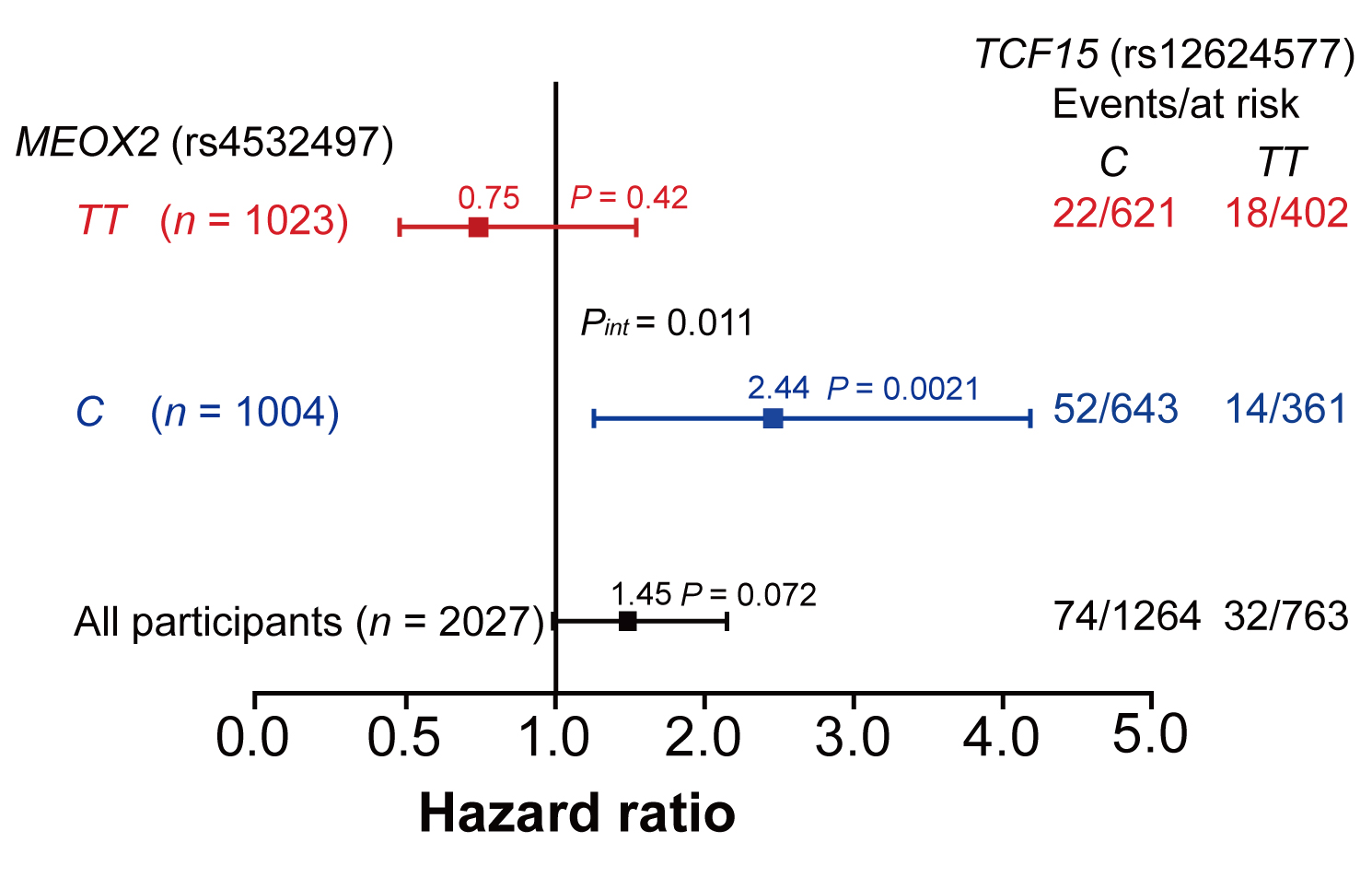


**Figure S2**

Plot of the *TCF15* gene and flanking regions on chromosome 20 (p13). The x‑axis represents the physical position on the chromosome (build 37, hg19). The y‑axis and the line indicate the recombination rate. The four framed SNPs with call rate ≥0.98 were retained in the analysis.


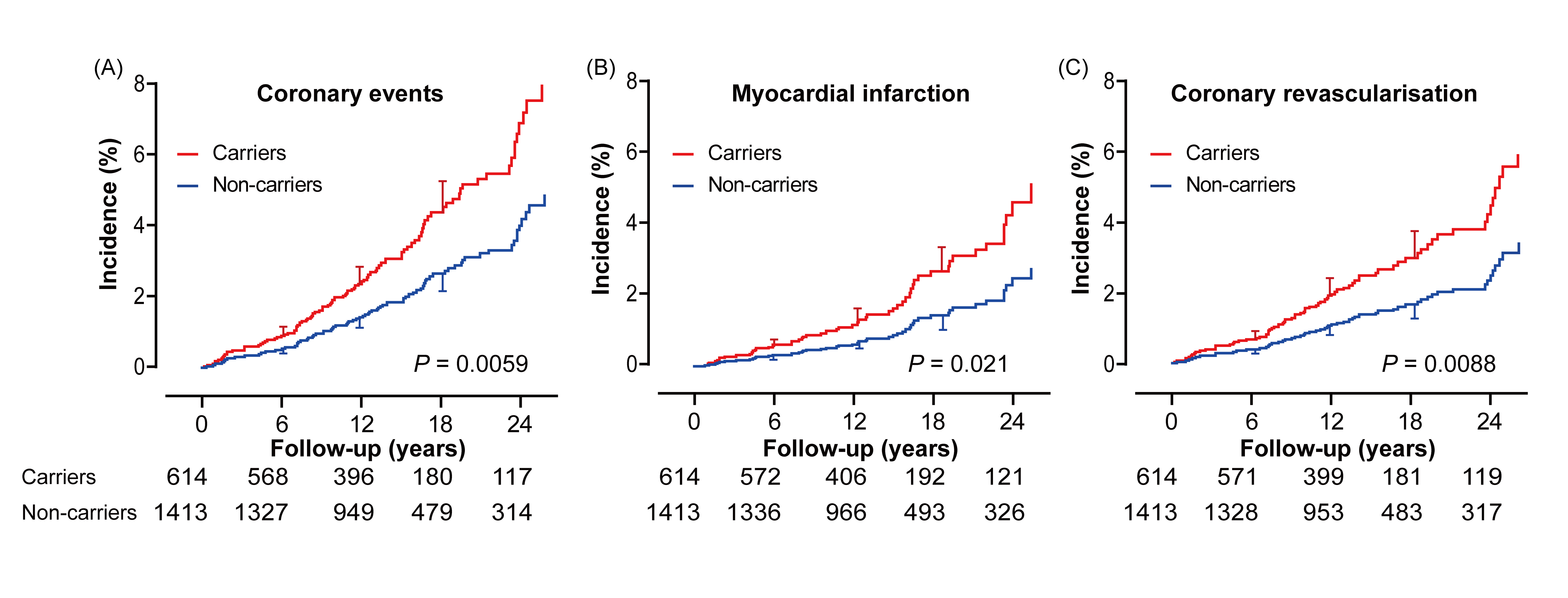


**Figure S3**

CHD risk in *TCF15* rs12624577 *C* carriers *vs.* *TT* homozygotes in three groups: *MEOX2* rs4532497 *C* carriers (*n* = 1004), *MEOX2* rs4532497 *TT* homozygotes (*n* = 1023), and all participants (*n* = 2027). Tabulated data are the number of participants with a CHD endpoint and the number at risk. Hazard ratios account for family clusters and were adjusted for baseline characteristics including sex, age, body mass index, systolic pressure, total-to-HDL cholesterol ratio, smoking and drinking, and antihypertensive drug treatment. Horizontal bars denote the 95% confidence interval. The *P*‑value for interaction was 0.011.

**Figure S4**

Incidence of coronary endpoints (A), myocardial infarction (B) and coronary revascularisation (C) in *MEOX2* *GTCCGC* carriers and non-carriers. Letters coding the haplotype refer to the rs10777, rs12056299, rs7787043, rs4532497 rs6959056 and rs1050290 alleles (see *Additional file 1: Tables S1* and *S2*). Haplotypes were reconstructed using the expectation-maximisation algorithm as implemented in the PROC HAPLOTYPE procedure of the SAS software version 9.3. Vertical bars denote the standard error. *P*‑values refer to the difference between non-carriers and carriers. Median follow-up was 15.2 years. Tabulated data are the number of participants at risk by *GTCCGC* carrying status at 6‑year intervals.
